# Burden of out-of-pocket expenditure for road traffic injuries in urban India

**DOI:** 10.1186/1472-6963-12-285

**Published:** 2012-08-28

**Authors:** G Anil Kumar, T Ramachandran Dilip, Lalit Dandona, Rakhi Dandona

**Affiliations:** 1 Public Health Foundation of India, ISID Campus, 4, Institutional Area, Vasant Kunj, New Delhi, 110 070, India; 2 Institute for Health Metrics and Evaluation, University of Washington, Seattle, WA, USA

**Keywords:** Costs, India, Out of pocket, Road traffic injuries

## Abstract

**Background:**

Road traffic injuries (RTI) are an increasing public health problem in India where out-of-pocket (OOP) expenditures on health are among the highest in the world. We estimated the OOP expenses for RTI in a large city in India.

**Methods:**

Information on medical and non-medical expenditure was documented for RTI cases of all ages that reported alive or dead to the emergency departments of two public hospitals and a large private hospital in Hyderabad. Differential risk of catastrophic OOP total expenditure (COPE-T) and medical expenditure (COPE-M), and distress financing was assessed for 723 RTI cases that arrived alive at the study hospitals with multiple logistic regression. Catastrophic expenditure was defined as expenditure > 25% of the RTI patient’s annual household income. Variation in intensity of COPE-M in RTI was assessed using multiple classification analysis (MCA).

**Results:**

The median OOP medical and non-medical expenditure was USD 169 and USD 163, respectively. The prevalence of COPE-M and COPE-T was 21.9% (95% CI 18.8-24.9) and 46% (95% CI 42–49.3), respectively. Only 22% had access to medical insurance. Being admitted to a private hospital (OR 5.2, 95% CI 2.7–9.9) and not having access to insurance (OR 3.8, 95% CI 1.9–7.6) were significantly associated with risk of having COPE – M. Similar results were seen for COPE - T. MCA analysis showed that the burden of OOP medical expenditure was mainly associated with in-patient days in hospital (Eta =0.191). Prevalence of distress financing was 69% (95% CI 65.5-72.3) with it being significantly higher for those reporting to the public hospitals (OR 2.8, 95% CI 1.7-4.6), those belonging to the lowest per capita annual household income quartile (OR 7.0, 95% CI 3.7-13.3), and for those without insurance access (OR 3.4, 95% CI 2.0-5.7).

**Conclusions:**

This paper has outlined the high burden of out-of-pocket medical and total expenditure associated with RTI in India. These data reinforce the need for implementing more effective financial protection mechanisms in India against the high out-of-pocket expenditure incurred on RTI.

## Background

Road traffic injuries (RTI) are a global problem affecting all regions of the world [1,2]. It is estimated that the annual cost of RTI in the low- and middle-income countries ranges between 1 to 3% of their GDP [1,3]. RTI are a major cause of mortality and morbidity in India [4,5] and burden of RTI in India has been rising over the past 2 decades, [6] with an estimated 1.2 million hospitalisations and 6 million non-hospitalised treatments in India due to RTI [5]. Even though RTI are increasingly contributing to the burden of disease in India, yet little is known about the economic consequences associated with RTI. Such consequences are important to understand in addition to the clinical consequences of RTI to fully appreciate the extent of burden of RTI. Estimating the cost of injuries is identified among five priority items to address global burden of unintentional injuries, most of which is accounted for by RTI [7].

The financial burden associated with RTI in India is likely to be significant. The public spending on health has remained low in India and the private out-of-pocket (OOP) expenditures on health is among the highest in the world, [8,9] thereby, resulting in the injured person’s household mostly bearing the financial burden of medical care in addition to the non-medical costs and associated wage losses. Impoverishment of households in India due OOP expenditure on health is well documented [9-12] and these are reported to be higher for injuries than for other ailments [13]. In this background, this paper documents financial burden of RTI in an urban population in India which could further strengthen the case for preventing RTI in India.

## Methods

### Study population

Ethics approval was obtained from the Administrative Staff College of India, Hyderabad, India, and the research conformed to the principles embodied in the Declaration of Helsinki. RTI cases were recruited for hospital and a follow up interview from two large public hospitals and three branches of a large private hospital in Hyderabad which cater to a significant proportion of RTI cases in the city. Study was conducted from November 2005 and June 2006. RTI cases of all ages that had either reported alive to the emergency department or were brought dead to these hospitals were included in the study. RTI was defined as any injury resulting from a road traffic crash (RTC) irrespective of outcome and severity. Trained interviewers were posted round-the-clock in the emergency department and mortuary to capture all RTI cases. Detailed methodology of this study is published elsewhere [14,15], and the details relevant to this paper are presented here.

Interviews were conducted using a questionnaire designed for this study after obtaining written informed consent from the injured person, care-taker or a responsible adult family member in case of death. Data was collected from the injured person wherever possible or from the care-taker or a responsible adult family member. Details of injuries sustained were completed by the hospital physician or the physician attached to the hospital mortuary. The injuries were classified according to broad categories as per International Statistical Classification of Diseases and related health problems Version 10 (ICD-10) [16] and the Abbreviated Injury Scale [17]. The Injury Severity Score (ISS) was derived for each case [18].

Detailed information was collected on demographics of the injured, characteristics of crash, and detailed costs of these RTI including access to insurance for RTI expenses. Information on a variety of medical and non-medical expenditure incurred due to RTI was documented on a daily basis by interviewers until each RTI patient was discharged alive or dead from the hospital. This information post discharge/death was also collected in a follow up interview held on an average after 6 months from date of discharge/death. Reimbursements which the RTI patients were able to claim from the employer, insurance company or from the other party involved in crash were also recorded. In cases where a patient had also sought medical care on more than one occasion, out of pocket expenses for each episode of treatment relating to the particular RTI were recorded and aggregated. *Medical expenditure* included all expenditure towards consultation, diagnostics, medicines, surgery, hospital charges, autopsy charges, rehabilitation/ physiotherapy, ambulance cost, and medical care after discharge from hospital. *Non-medical expenditure* comprised of expenditure on food, phone, transportation of family/caretakers, repair of vehicle, legal expenses, compensation paid to the other party involved in crash, costs of obtaining death certificate, funeral and bribes paid (if any).

### Data analysis

Data were entered in an MS Access database and SPSS version 17 (SPSS Inc. Chicago, IL, USA) was used for statistical analysis. The main aim of this analysis was to examine the risk and intensity of catastrophic OOP total and medical expenditure due to RTI. We defined catastrophic expenditure as expenditure >25% of the RTI patient’s annual household income. The monthly household income at the time of RTC was annualized for this computation, which included household income for all members of the household from all sources including salary/wages, income from rents, royalties on leased lands or properties, interests, and income from farming, livestock etc. We have used net OOP expenditure for analysis after deducting reimbursements for RTI expenses from the total OOP expenses. The analysis also extends to understand the level of distress financing in the case of RTI where distress financing was defined as financial activities undertaken by the household as a result of RTI including borrowing money from relatives/ friends, taking loan from banks/other lenders, or selling assets.

Of the 781 RTI cases recruited for this study, 741 (94.9%) had arrived alive and remaining 40 cases were dead on arrival at the study hospitals. Of the 741 cases that had arrived alive, follow-up interview was completed for 723 (97.6%) cases. Among the 40 cases that had arrived dead, follow-up interview was completed for 34 (85%) cases. Six of these 40 dead on arrival cases had incurred medical expenditure on RTI with another health care provider prior to arriving dead at the study hospitals; the medical costs incurred by these respondents is included in this analysis, and the type of hospital for them is the facility where the medical expenditure was incurred and not where these respondents were brought dead.

Descriptive out-of-pocket expenditure data are presented for all RTI cases that were followed up. Multiple logistic regression analysis was performed to examine the differential risk of catastrophic OOP total expenditure (COPE-T) and medical expenditure (COPE-M), and distress financing for 723 RTI cases who had arrived alive at the study hospitals with covariates including the type of road user, type of hospital, per-capita annual household income quartiles, duration of hospitalisation, severity of RTI, and access to insurance. The selection of co-variates was based on the previously published literature within the Indian context and of all the possible co-variates that were explored, the ones which had significant association (p-value <0.25) with the outcome variables were used in the multivariate analysis. In the regression model, the effect of each category of a multi-categorical variable was assessed by keeping the first or the last category as reference, and all the variables were introduced simultaneously in the model. 95% confidence intervals (CI) for odds ratio are also reported. Multivariate analysis of the variation in intensity of COPE-M in RTI was performed using multiple classification analysis (MCA) for these cases [19]. The ratio of OOP medical expenditure to annual household income of the RTI patient was computed as the dependent variable to examine the risk and intensity of COPE-M due to RTI. The interaction between income and type of hospital utilised for medical care was accounted for in all analyses. Severity of RTI was classified as *less severe* (ISS 1–4) and *severe* (ISS >4) for this analysis based on the median ISS of 4. All RTI cases that were brought dead to the hospitals were assigned the *severe* injury severity category.

## Results

The OOP expenditure incurred by the 723 RTI cases that arrived alive at the study hospitals is presented in Table 1. The median OP medical and non-medical expenditure was USD 170 and 162, respectively. Median medical expenditure was >4 times higher in the private hospitals as compared with that in public hospitals. However, median non-medical expense was higher in public hospitals (USD 170) than in the private hospital (USD 130). The median OOP medical expenses ranged from USD 115 in the poorest income quartile to USD 360 in the richest income quartile with this difference between the two quartiles being statistically significant.

**Table 1:** Mean, median and range for the out-of-pocket expenditure incurred for those who arrived alive at study hospitals following road traffic injury by select variables

**Variable**	**Category**	**N**	Medical expenditure* (In USD)			**Non-medical expenditure† (In USD)**			**Total expenditure ‡ (In USD)**		
			**Mean (SD)**	**Median**	**Range**	**Mean (SD)**	**Median**	**Range**	**Mean (SD)**	**Median**	**Range**
Type of road user§	Pedestrian/cyclist	220	379.8 (880.5)	118.8	0-9,322.4	257.1 (290.3)	151.8	4.2- 1,636.4	636.9 (950)	341.4	5.8- 9,710.8
	MTV	356	642.7 (1345.9)	262.7	0-17,498.0	349.5 (755)	166	3.7-10,472.7	992.1 (1716.7)	573.0	20.6- 1,716.7
	Others	147	390.1 (577.4)	143.9	0-3,501.1	324.5 (646.6)	167	4.1 -5,405.3	714.6 (968.2)	398.3	17.7- 5,912.8
Type of hospital**	Private		1095 (1775)	533	9-17,498	416.8 (1108)	127.3	3.7-10,472.7	1511.9 (2334.2)	800.8	71.4- 19,973.6
	Public	555	334.7 (701.9)	119.3	0-9,322.4	285.8 (369.9)	170.3	4.1- 5,405.3	620.5 (836.8)	377.8	5.8- 9,709.8
Per capita annual household income quartile††	I (lowest)	197	439.2 (1422.4)	114.8	0-17,498	304.7 (495.2)	171.3	4.8- 5,405.3	743.8 (1657.1)	345.2	11.5-19,973.6
	II	192	386.9 (818.6)	142.6	0-7,096.3	311.1 (606.7)	192.5	4.1- 7,663.6	698 (1088.2)	389.3	17.7- 8,085.2
	III	153	376.6 (489.3)	165.1	0-3,062.5	253.8 (288.1)	136.8	3.7- 1,473	630.3 (608)	421.9	5.8- 3,560.5
	IV (highest)	180	839.7 (1264.8)	360.6	0-9,322.4	386.9 (916.9)	138.3	7.2- 10,472.7	1226.6 (1737.5)	722.4	10.0-14,451.1
Total		723	511.3 (1100)	169.3	0- 17,498	316.3 (626.1)	162.5	3.7-10,472.7	827.6 (1392.6)	446.5	5.8 19,973.6

SD refers to standard deviation.

*Medical expenses paid at the hospital including the expenses for ambulance services.

†Non-medical expenses include transport expenses other than those for ambulance, food and phone expenses incurred by the household related to this road traffic crash (RTC), repair/damage expenses of vehicle involved in RTC, legal and other miscellaneous expenses.

‡Sum of medical and non-medical expenses.

¶USD: United States Dollar; 1 USD was approximately Indian Rupees 44 during the study period.

§Kruskal test for equality of median: p = 0.614, 0.476 and <0.001 for medical, non-medical and total expenditure respectively; MTV: motorosied two-wheeled vehicles including moped/luna, scooter/scootertte and motorcycle; others include all vehicles other than MTV/ cycle & pedestrians.

**Kruskal test for equality of median: p = 0.084, 0.537 and <0.001 for medical, non-medical and total expenditure respectively.

††Data not available for 1 participant; Kruskal test for equality of median: p = 0.001, 0.041 and <0.001 for medical, non-medical and total expenditure respectively.

The detailed components of OOP non-medical expenditure are described in Table 2. The median OOP expenditure for food and phone was high (USD 52) followed by transportation expenses (USD 50). The expenses for transportation and food and phone were significantly higher for RTI cases that were treated in public hospitals than in those in the private hospital (p < 0.001), while the miscellaneous expenditure was significantly higher in the former (p < 0.001). Those in the richest per capita income quintile spent significantly less than those in the lower quintiles for transportation, food and phone, and miscellaneous items.

**Table 2:** Mean, median and range for the components of non-medical expenditure incurred for those who arrived alive at study hospitals following road traffic injury by select variables

**Variable**	**Category**	**N**	**Type of cost**														
			Transportation* (In USD**)			Food & phone† (In USD**)			Vehicle‡ (In USD**)			Legal (In USD**)			Miscellaneous§ (In USD**)		
			**Mean (SD)**	**Median**	**Range**	**Mean (SD)**	**Median**	**Range**	**Mean (SD)**	**Median**	**Range**	**Mean (SD)**	**Median**	**Range**	**Mean (SD)**	**Median**	**Range**
Type of road user††	Pedestrian/ cyclist	220	84.6	56.5	1.6-84.1	72.8	54.5	0.8-359.8	0.5	0	0-18.2	7.3	0	0-134.1	91.8	5	0-1,386.4
			(103.6)			(64)			(2.1)			(19.7)			(236.7)		
	MTV	356	107.1	42.7	0.8-8,015.9	83.2	47.6	0.6-1,688.3	49.7	5.1	0-2,181.8	11.2	0	0-545.5	98.2	4.3	0-4,697.7
			(453.8)			(118.8)			(142.5)			(42.2)			(349)		
	Others	147	70	54.8	0.3-316.7	81.1	58.2	3.0-732.4	102.2	0	0-4,545.5	27.1	0	0-2,386.4	44.2	3.1	0-686.4
			(59.1)			(82.7)			(516.4)			(200.1)			(119.3)		
Type of hospital‡‡	Private	168	135.2	34.3	0.8-8,015.9	74.6	39	0.6-1,688.3	79.3	0	0-3,409.1	22.4	0	0-2,386.4	105.3	1.1	0-4,697.7
			(654.7)			(147.1)			(338.9)			(185.3)			(446)		
	Public	555	79.9	56.8	0.3-700.2	81.2	58.2	0.8-732.4	35.1	0	0-4,545.5	10.5	0	0-545.5	79.2	5.7	0-1,386.4
			(86.8)			(77.3)			(223.4)			(39.2)			(210.7)		
Per capita annual household income quartile‡‡ $	I (lowest)	197		59.1	0.8-686.4		52.6	1.0-1,688.3		0	0-4,545.5		0	0-545.5		5	0-1,386.4
			72.1			85			43			10.8			93.2		
			(71.7)			(139.7)			(327.8)			(42.2)			(246.1)		
	II	192	101.2	62.7	0.3-2,597.7	85.2	69.8	3.0-464.0	35.2	0	0-1,818.2	7.7	0	0-204.5	81.3	6	0-4,697.7
			(204)			(71.8)			(172.1)			(23.2)			(361.6)		
	III	153	76.7	42.5	1.4-840.9	70	46.4	0.8-336.8	28.7	0	0-544.0	9.1	0	0-417.0	68.7	3	0-1,250.9
			(109)			(64.8)			(85)			(37.5)			(211.1)		
	IV (highest)	180	120.1	35.8	1.1-8015.9	76.2	43.2	0.6-653.8	73.4	0	0-3,409.1	25.5	0	0-2,386.4	93.6	1.8	0-2,275.0
			(603.1)			(90)			(326.4)			(181.5)			(281.2)		
Total		723	92.7	50.6	0.2-8015.9	79.6	52.3	0.6-1,688.3	45.4	0	0-4,545.5	13.3	0	0-2,386.4	85.3	4.3	0-4,697.7
			(324.7)			(97.9)			(255.3)			(95.6)			(283.2)		

SD refers to standard deviation.

* All transport expenses other than ambulance.

† Food and phone expenses incurred by the household related to this road traffic crash (RTC).

‡ Repair/damage expenses of vehicle involved in RTC.

**¶** Expenses related to police, compensation, death certificates, and lawyers to this RTC.

§ Expenses other than transportation, food/phone, vehicle and legal costs.

**United States Dollar; 1 USD was approximately Indian Rupees 44 during the study period.

†† Kruskal test for equality of median: p = 0.082, 0.493, , and 0.404 for transport, food & phone, and miscellaneous expenditure respectively; MTV: motorosied two-wheeled vehicles including moped/luna, scooter/scootertte and motorcycle; others include all vehicles other than MTV/ cycle & pedestrians.

‡ ‡ Kruskal test for equality of median: p < 0.001 for transport, food & phone, and miscellaneous expenditure.

**¶¶** Data not available for 1 participant; Kruskal test for equality of median: p = 0.008, 0.040, and 0.003 for transport, food & phone, and miscellaneous expenditure respectively.

Data on total OOP expenditure incurred by 34 RTI cases that were dead on arrival at the study hospitals is presented in Table 3. The median total OOP expenditure for these cases was USD 985 which ranged from USD 660 for the lowest per capita income quartile to USD 1,245 for the highest income quartile.

**Table 3:** Mean, median and range for the out-of-pocket expenditure incurred for those brought dead to the study hospitals following road traffic injury by select variables

**Variable**	**Category**	**N**	Total out-of-pocket expenditure * (In USD†)		
			**Mean (SD)**	**Median**	**Range**
Type of road user‡	Pedestrian/cyclist	10	1114 (917.4)	770.5	407.3-3,475
	MTV	14	1453.4 (751.4)	1,263.1	375.0-3,117
	Others	10	793 (231.8)	797.2	482.7-1,188.6
Per capita annual household income quartile	I (lowest)	8	1025 (900.5)	661.9	375.0-3,117
	II	9	948.5 (343.9)	820.5	663.6-1,735.2
	III	6	1057 (626.3)	834.7	407.3-1,963.6
	IV (highest)	11	1485.4 (872.4)	1,246.6	486.4-3,475
Total		34	1159.4 (738)	986	375.0-3,475

SD refers to standard deviation.

*Transport expenses, food and phone expenses incurred by the household related to this road traffic crash (RTC), repair/damage expenses of vehicle involved in RTC, legal and other miscellaneous expenses.

†United States Dollar; 1 USD was approximately Indian Rupees 44 during the study period.

‡ Kruskal test for equality of median: p = 0.045 for total out-of-pocket expenditure; MTV: motorosied two-wheeled vehicles including moped/luna, scooter/scootertte and motorcycle; others include all vehicles other than MTV/ cycle & pedestrians.

¶ Kruskal test for equality of median: p = 0.197.

Among the 723 RTI cases that had arrived alive, 158 (21.9%, 95% CI 18.8-24.9) and 330 (46%; 95% CI 42–49.3) had COPE - M and COPE – T, respectively. On applying multiple logistic regression (Table 4), increasing in-patient days in hospital was significantly associated with risk of having COPE – M with >7 in-patient days having the highest odds (odds ratio, OR 10.5, 95% CI 5.9–19.0) followed by those admitted in a private hospital (OR 5.2, 95% CI 2.7–9.9). The odds of having COPE – M increased with decreasing per capita annual income quartiles, and access to insurance also showed significant association with it (OR 3.8, 95% CI 1.9–7.6). The results of multiple logistic regression for COPE - T were similar to that for COPE - M except that ISS >4 was significantly associated with COPE – T (OR 2.1, 95% CI 1.5–3.0). Also, the results of multiple logistic regression models for having OOP medical and total expenditure >30% of annual household income were similar to that for COPE – M and COPE - T (data not shown).

**Table 4:** Multiple logistic regression analysis for catastrophic out-of-pocket medical and total expenditure for those who arrived alive at study hospitals following road traffic injury

**Variable**	**Category**	Total N = 722*	**Out-of-pocket medical expenditure**^†^		**Out-of-pocket total expenditure**^†^	
			**Number with catastrophic expenditure**	**Odds ratio for catastrophic expenditure (95% CI)**	**Number with catastrophic expenditure**	**Odds ratio for catastrophic expenditure (95% CI)**
Type of road user‡	Pedestrian/cyclist	220	38 (17.3)	0.6 (0.3-1.0)	106 (48.2)	0.9 (0.6-1.5)
	MTV	356	86 (24.2)	1.2 (0.7-2.0)	160 (44.9)	1.4 (0.9-2.2)
	Others	146	34 (23.3)	1	67 (45.9)	1
Type of hospital	Private	168	40 (23.8)	5.2 (2.7-9.9)	56 (33.3)	2.0 (1.2-3.5)
	Public	554	118 (21.3)	1	277 (50.0)	1
Per capita annual household income quartile§	I	197	67 (34.0)	4.6 (2.3-9.0)	133 (67.5)	7.3 (4.1-13.0)
	II	192	39 (20.3)	1.8 (0.9-3.6)	98 (51.0)	3.2 (1.8-5.4)
	III	153	25 (16.3)	1.6 (0.8-3.2)	59 (38.6)	2.4 (1.4-4.1)
	IV	180	27 (15.0)	1	43 (23.9)	1
In-patient days§	<4	266	20 (7.5)	1	80 (30.1)	1
	4 - 7	140	19 (13.6)	2.4 (1.2-4.8)	47 (33.6)	1.3 (0.8-2.2)
	> 7	316	119 (37.7)	10.5 (5.9-19.0)	206 (65.2)	4.6 (3.0-6.8)
Injury severity§**	1-4	379	62 (16.4)	1	131 (34.6)	1
	>4	330	92 (27.9)	1.2 (0.8-1.8)	198 (60.0)	2.1 (1.5-3.0)
Access to insurance§	Yes	153	16 (10.5)	1	32 (20.9)	1
	No	569	142 (25.0)	3.8 (1.9-7.6)	301 (52.9)	3.3 (1.9-5.6)

Catastrophic expenditure is defined as >25% of the annual household income of the injured. CI refers to confidence interval.

*Data missing for 1 case due to non-reporting of annual household income.

†Omnibus test for model coefficients: p < 0.0001; Nagelkerke R Square: 30% and 33%; and Hosmer and Lemeshow χ2 p = 0.96 and 0.27 for out-of-pocket medical and total expenditure, respectively.

‡MTV: motorised two-wheeled vehicles including moped/luna, scooter/scootertte and motorcycle; others include all vehicles other than MTV/ cycle & pedestrians.

**¶**χ2 test for significance: p < 0.001 for out of pocket medical expenditure.

§χ2 test for significance: p < 0.001 for out of pocket total expenditure.

**Data not available for 14 participants.

MCA of the variation in intensity of burden associated with out-of-pocket medical expenditure for 723 RTI cases that had arrived alive at the study hospitals is presented in Table 5. The unadjusted mean of ratio of the medical cost to total annual household income was 0.241 for pedestrians, indicating that on an average, OOP medical expenditure for RTI as a pedestrian was about 24.1% of the injured person’s per capita annual household income. The eta values presented for unadjusted means show that burden of OOP medical expenditure to total annual household income was mainly associated with in-patient days in hospital (Eta =0.191), followed by per capita annual household income, injury severity, type of hospital, and type of road user. Average expenditure level was 39% for RTI cases treated in private hospitals as compared with 22% in public hospitals. Burden of medical expenditure was nearly 3 times higher in the lowest per capita annual household income quartile (45%) than among the highest income quartile (16%). Unadjusted mean value of expenditure burden increased with in-patient days and severity of injury. Large variations between adjusted and unadjusted means were noticed based on the type of hospital, with OOP expenditure equivalent to more than 100% of household annual income in a private hospital as compared with about 1% in public hospital. The second most important correlate of intensity of OOP medical expenditure was number of in-patient days (Beta = 0.190), where differences in means remained nearly similar after controlling for the other variables. Effect of type of road user and severity of injury was insignificant, when the effect of other covariates were held constant.

**Table 5:** Multiple classification analysis of intensity of catastrophic medical expenditure for those who arrived alive at study hospitals following road traffic injury

**Variable**	**Category**	Total N = 722*	**Predicted mean out-of-pocket medical expenditure to household annual income ratio**				
			**Unadjusted mean**		**Adjusted mean†**		
			**Mean**	**Eta**	**Mean**	**Beta**	**P value**
Type of road user‡	Pedestrian/cyclist	216	0.241	0.050	0.206	0.061	0.255
	MTV	348	0.297		0.313		
	Others	145	0.177		0.189		
Type of hospital	Private	164	0.386	0.077	1.072	0.479	<0.001
	Public	545	0.216		0.010		
Per capita annual household income quartile	I (lowest)	192	0.454	0.136	0.495	0.160	0.001
	II	190	0.242		0.208		
	III	150	0.134		0.160		
	IV (highest)	177	0.158		0.126		
Number of in-patient days	<4	262	0.087	0.191	0.073	0.190	<0.001
	4 - 7	137	0.120		0.154		
	> 7	310	0.458		0.454		
Injury severity	1-4	379	0.156	0.113	0.212	0.050	0.187
	>4	330	0.369		0.305		
	Full model	723				0.359	<0.001

*Data missing for 1 case due to non-reporting of annual household income.

†F test value 8.6 and significance <0.001; Goodness of fit: 14%.

‡MTV: motorised two-wheeled vehicles including moped/luna, scooter/scootertte and motorcycle; others include all vehicles other than MTV/ cycle & pedestrians.

**¶**Data not available for 14 participants.

A total of 498 (69%, 95% CI 65.5-72.3) of the RTI cases that had arrived alive reported distress financing to cover the expenses related to RTI (Table 6). The odds of distress financing were significantly higher for those treated in the public hospitals (OR 2.8, 95% CI 1.7-4.6) than in private hospitals, and was 7 times higher (95% CI 3.7-13.3) for those belonging to the lowest per capita annual household income quartile as compared with the highest income quartile. Similar to the catastrophic expenditure, the risk of distress financing significantly increased with increasing in-patient days and severity of RTI. An RTI patient without insurance access was 3.4 times (95% CI 2.0-5.7) more likely to be at the risk of distress financing than the counterpart with insurance access.

**Table 6:** Results for multiple logistic regression analysis of distress financing for those who arrived alive at study hospitals following road traffic injury (RTI)

**Variable**	** Category**	** Total N = 723**	Distress financing*	
			**Number with distress financing (%)**	**Odds ratio for incurring distress financing (95% CI)**
Type of road user^†^‡	Pedestrian/cyclist	220	178 (80.9)	1.4 (0.8-2.6)
	MTV	356	215 (60.4)	0.9 (0.5-1.5)
	Others	147	105 (71.4)	1
Type of hospital‡	Private	168	53 (31.5)	1
	Public	555	445 (80.2)	2.8 (1.7-4.6)
Per capita annual household income quartile‡	I (lowest)	197	175 (88.8)	7.0 (3.7-13.3)
	II	192	154 (80.2)	3.7 (2.1-6.5)
	III	153	104 (68.0)	3.2 (1.8-5.6)
	IV (highest)	180	65 (36.1)	1
In-patient days‡	<4	266	148 (55.6)	1
	4 - 7	140	81 (57.9)	1.3 (0.8-2.3)
	> 7	317	269 (84.9)	4.5 (2.8-7.4)
Injury severity‡§	1-4	380	227 (59.7)	1
	>4	330	262 (79.4)	1.8 (1.1-2.6)
Access to insurance‡	Yes	153	50 (32.7)	1
	No	570	448 (78.6)	3.4 (2.0-5.7)

CI refers to confidence interval.

* Omnibus test for model coefficients p < 0.001; Nagelkerke R Square 46%, and Hosmer and Lemeshow χ2 p = 0.09.

†MTV: motorised two-wheeled vehicles including moped/luna, scooter/scootertte and motorcycle;

others include all vehicles other than MTV/ cycle & pedestrians.

‡χ2 test for significance: p < 0.001.

Data missing for 1 participant.

§Data not available for 14 participants.

## Discussion

RTI constitute a major health burden in India, however, efforts to address this burden have been hampered by the lack of a multi-sectoral coordinated approach and inadequacy of data on various aspects of RTI burden including the associated costs [20]. This paper has outlined the high burden of out-of-pocket medical and total expenditure associated with RTI in India which is increasingly characterized by disproportionate increase in the number of motorised vehicles in comparison with the expansion of road network [21]. To the best of our knowledge, this is the first comprehensive attempt to explore the details of OOP expenses for RTI in urban India. Hospital-based recruitment of RTI cases including fatal and non-fatal cases of varying severity from public and private sector hospitals in this study has provided a wide ranging perspective on the costs associated with RTI, and can be considered representative of the cases that report to large hospitals. Daily documentation of expenditure for each RTI case during the hospital stay by the interviewers, thereby, reducing recall bias, is a major strength of this study. As highlighted by these data, a patient seeking medical care for RTI in India faces the consequence of being in a health system noted for incurring high OOP payments in both public and private hospitals, and for a higher risk of impoverishment at household level due to RTI-related spending [10,11].

The ratio of OOP medical expenditure to annual household expenditure has been widely used to determine catastrophic expenditure due to ill health. A wide range of thresholds have been used to define catastrophic expenditure ranging from 10% to 40% of the household expenditure [11,22-24]. The capacity to pay or non-subsistence spending capacity has also been used instead of the total household expenditure to estimate catastrophic expenditure relating to medical care [25]. In this study, we did not collect data on household expenditure and hence used the annual household income to determine the nature of catastrophic expenditure using a threshold of 25% of the annual household income. This is in the middle of the 10-40% range used in the literature, which seems reasonable for the urban context in India. We did this analysis using a threshold of >20% and >30% of the annual household income, and the results of multiple logistic regression were similar. In addition, we also examined the intensity of catastrophic payment with another approach using multiple classification analysis.

The average OOP medical expenditure for RTI in this study was 2.5 times higher than the average medical expenditure per hospitalisation reported from urban India during the same period, [26] suggesting a relatively higher adverse impact of OOP expenditure due to RTI on a household as compared with other illnesses. Added to this medical burden is the high burden of non-medical expenditure in RTI which is nearly similar to the average medical expenditure for hospitalisation due to any illness in India [26]. Thus, these data suggest that a large proportion of those suffering RTI are incurring a double burden of high medical and non-medical expenses, thereby making households quite vulnerable to catastrophic OOP in RTI. Transportation, food and phone expenses were the major items in the non-medical expenditure category with the cost of vehicle damage the next important category of expenditure. It is possible that the catastrophic expenditure due to RTI is underestimated in these findings as it is likely that some patients would have continued to incur RTI expenditure beyond the follow-up period of this study. However, it is important to note that as these expenditure figures for RTI cases include costs 6 months beyond the crash they are more complete as compared with costs incurred only during the hospital admission/visit. On the other hand, since incomes are often underreported in household surveys, our catastrophic expenditures could be an overestimate for this reason. The absence of overall household expenditure data is a limitation of our study.

This analysis also highlights the significant role of public hospitals in providing RTI related medical care services. This inference is based on MCA findings that clearly show the public-private divide in OOP medical expenses for RTI widens drastically when other variables are controlled for. However, it cannot be discounted that the injured treated in public hospitals also spend a substantial share of their household income. Distress financing was also significantly high for those treated in public hospitals. To curtail such expenses in the public health system, it needs to be well-equipped in terms of drugs, supplies and diagnostics, [27] as these are reported to constitute over 95% of OOP expenses in public hospitals [28]. In addition, our study has also highlighted that more severe RTI cases are brought to public hospitals than private hospitals with the mean injury severity score being 37 and 30 for those treated in former and latter, respectively. Therefore, these data also provide evidence for the general notion that the private hospitals are engaged in a limited manner in treatment of RTI but involve high household OOP expenditure as compared with the public hospitals. Another notable finding was the long duration of hospitalisation in case of RTI which further exaggerate OOP expenditure with nearly 43% of RTI cases in this study remaining hospitalised for more than 7 days. These data provide further evidence for the need to strengthen public health system in India in order to reduce OOP expenditure on health care and a well regulated integration of the private sector within the national health-care system [29].

Only 22% of the injured had access to some form of insurance or reimbursement to cover a part of / total RTI expenses. Even though these data are 5-years old, the current health insurance coverage at household level by any scheme is only 10% in urban India [30]. The subgroup with insurance access had a much lower risk of having catastrophic expenditure and also had a lower chance of distress financing for seeking care for RTI. Despite it being mandatory to have at least third party insurance for motorized vehicles in India, there is a gross violation of this element in practice. In this study sample, only 34% and 8% of those travelling in motorized vehicles at the time of RTC reported that vehicle to have full insurance and third part insurance, respectively. Effective implementation of insurance for the motorized vehicles may be useful in reducing the incidence of such catastrophic spending due to RTI.

As expected, those belonging to the richest annual household income quartile despite incurring a higher level of OOP expenditure on RTI had a lower household level burden as compared with those in the poorest income quartile. Assuming that those belonging to the lowest household income quartile incur expenditure based on the concept of subsistence expenditure, [25] our data suggest that on average this group spends about 50% of their annual household income for RTI medical care and is 7 times more likely to have distress financing for RTI care. Those with RTI cases from higher income quartiles especially the richest have better access to insurance cover, thereby helping them in reducing the quantum of household OOP expenditure. Hence the impoverishment effect of RTI is the most severe on the poorest.

A government insurance initiative in India, the Rashtriya Swastha Bima Yojana (RSBY) rolled out in 2008, is expected to protect the below poverty line households from financial liabilities arising out of medical care for ill health of household members by providing up to USD 675 for inpatient medical care per household annually [31]. If we consider in our study those under per capita income quartile group I as being below the poverty line, [32] 34% of them had COPE-M with 38.8% among these having medical expenditure ≥ USD 675. In this background, even if access to RSBY were made universal among the below poverty line households, 28% of these households would have still have had COPE – M. These findings suggest the RSBY scheme should find ways to better target those who are vulnerable to catastrophic health expenditure. In addition, these data provide further evidence to support the increasing calls for universal health care in India by 2020 to provide optimum benefit to people who bear a disproportionate burden of disease and health care [29].

There is increasing evidence of the substantial financial burden associated with RTI from various parts of the world, [33-37] reinforcing the need for implementation of evidence-based and cost-effective strategies to reduce the burden of RTI as is also evident from the data presented in this paper [7,38]. These data also highlight that the non-medical expenses contribute equally to the burden of RTI as the medical expenses, [39] thus making it necessary to consider these in understanding the total burden of RTI on households and on society at large.

## Conclusion

This paper has outlined the high burden of out-of-pocket expenditure associated with RTI in urban India. In the background of high RTI related mortality and morbidity, these high burden estimates of out-of-pocket expenditure provide further impetus to enhance road safety in India. The inequitable financial burden of RTI including distress financing highlight the need to better target the population groups most vulnerable by improving access to insurance and universal health care in order to reduce the out-of-pocket expenditure burden of RTI in India.

## Competing interests

The authors declare that they have no competing interests.

## Authors’ contributions

GAK led the analysis and prepared the first draft of manuscript. DTR contributed to the analysis, interpretation and manuscript writing. LD contributed to the design, interpretation and manuscript writing. RD conceived this study, led the design, analysis and interpretation, and drafted the final manuscript. All authors approved the final version of the manuscript.

## Funding source

This study was supported by the Wellcome Trust, UK (Grant 077002/Z/05/Z).

## Pre-publication history

The pre-publication history for this paper can be accessed here:

http://www.biomedcentral.com/1472-6963/12/285/prepub
